# Comparative analysis of rhizosphere microbial communities and secondary metabolites in cultivated *Rheum officinale* from different regions of China

**DOI:** 10.3389/fpls.2025.1650792

**Published:** 2025-09-30

**Authors:** Yan Wang, Feng Yan, Yujie Liang, Xiaochen Hu, Jing Gao, Mingying Zhang, Yonggang Yan, Gang Zhang, Yimin Li

**Affiliations:** ^1^ Key Laboratory for Research and Development of “Qin Medicine” of Shaanxi Administration of Traditional Chinese Medicine, Shaanxi University of Chinese Medicine, Xi’an, China; ^2^ College of Pharmacy and Shaanxi Qinling Application Development and Engineering Center of Chinese Herbal Medicine, Shaanxi University of Chinese Medicine, Xi’an, China; ^3^ State Key Laboratory of Research and Development of Characteristic Qin Medicine Resources (Cultivation), Shaanxi University of Chinese Medicine, Xianyang, China

**Keywords:** 16S rRNA sequencing, fungal ITS high-throughput sequencing, *Rheum officinale* Baill., secondary metabolites, soil properties

## Abstract

*Rheum officinale* Baill., a medicinal herb rich in anthraquinones and tannins, exhibits region-specific variation in bioactive compound accumulation. Given the well-documented pharmacological properties of *R. officinale* Baill. including its laxative, antibacterial, anti-inflammatory and regulating intestinal function, this study integrated Illumina sequencing of rhizosphere microbiomes, HPLC quantification of 10 active components (e.g., rhein, physcion), and soil analysis across three cultivation regions Shaanxi (ZB), Hubei (HB), Chongqing (CQ). Results demonstrated significant regional disparities: ZB showed highest rhein and catechin levels, while CQ accumulated more physcion. Microbial diversity followed the order ZB>HB>CQ, with *Proteobacteria* and *Ascomycota* dominating bacterial and fungal communities, respectively. Soil pH, moisture (SWC), and Zn/Cu content strongly correlated with microbial structure. Notably, *Rokubacteriales* was significantly positively associated with anthraquinones accumulation. These findings suggest that soil properties modulate microbial communities, which in turn regulate secondary metabolite biosynthesis through nutrient cycling (e.g., nitrogen/phosphorus metabolism). This study elucidates the tripartite interaction of soil-microbe-metabolite networks in *R. officinale* Baill., providing insights for geoherbalism optimization. Future research will focus on optimizing *R. officinale* Baill. cultivation through soil condition management to enhance both quality and yield.

## Introduction

*Rheum officinale* Baill. as a tall perennial herbaceous plant, its dried roots and rhizomes are known as the Chinese herbal medicine rhubarb (Chinese Pharmacopoeia Commission, 2025). The main chemical constituents include anthraquinones, anthrones, stilbenes, phenylbutanones, polysaccharides, tannins, and volatile oils (Cao et al., 2017). The laxative activity of *R. officinale* Baill. is primarily attributed to its anthraquinones derivatives (rhein, aloe-emodin) and anthrone glycosides (sennoside) (Duval et al., 2016; Takayama et al., 2012; Feng et al., 2013). The growing medicinal demand for *R. officinale* Baill. has outpaced wild supply, necessitating large-scale cultivation. While intensive farming system are now established in China’s major production regions, persistent monoculture challenges have emerged. Notably, soil-borne diseases (particularly root rot) have increased substantially due to continuous cropping issues, threatening industry sustainability (Lin et al., 2024; Xu et al., 2025). Research confirms these agricultural problems correlate strongly with soil microbiome alterations, where pathogenic bacterial/fungi and nutrient imbalances directly impact plant health (Pang et al., 2022; Bi et al., 2023). Beneficial secondary metabolites (such as medicinal plant active ingredients, microbial functional compounds, plant polyphenols, etc.) have exploded in market demand in recent years due to their high application value in medicine, food and health care, etc.; The contradiction between insufficient adaptation of production scale and demand expansion is continuing to give birth to deepening basic research in this field.

Rhizosphere microorganisms are often termed the plant’s ‘second genome’. In natural environment, plant roots harbor a diverse community of microorganisms within and surrounding the root system, collectively known as the rhizosphere microbiome, which includes bacteria and fungi (Luo et al., 2024). This microbiome accompanies plants throughout their life cycle, interacts closely with them, and plays a vital role in the growth, development, and secondary metabolite accumulation of medicinal plants (Yuan et al., 2022; Peng et al., 2020). Among these microorganisms are plant growth-promoting rhizobacteria (PGPR). PGPR constitute a core functional group that directly or indirectly sustain plant growth and soil health through mechanisms such as promoting nutrient uptake, secreting plant hormones, enhancing tolerance to abiotic stresses, and suppressing pathogenic microorganisms (Jabborova et al., 2024; Vaghela and Gohel, 2023). Certain rhizobacteria directly promote plant growth by secreting plant hormones or influencing the plant’s own hormone synthesis. Among them, phosphate-solubilizing bacteria (*Bacillus*, *Pseudomonas*, *Enterobacter* and *Escherichia*) represent the most dominant phosphorus-transforming groups in soil (Wang et al., 2023; Pang et al., 2024). Nitrogen-fixing bacteria such as *Klebsiella* and *Rhodococcus* can convert atmospheric nitrogen into ammonia, increasing soil nitrogen content and thereby promoting the growth of medicinal plants and the accumulation of their active ingredients (Shi et al., 2023). For instance, the latest research has found that *Paraburkholderia*, a probiotic flora in *Coptis chinensis*, can promote plant growth, activate immune responses, inhibit the main pathogens of coptis root rot, and significantly improve the overall health of plants (Cao et al., 2025), while *Streptomyces* TM32 from the rhizosphere of *Curcuma longa* exhibits potent antimicrobial effects against plant pathogens (Nakaew et al., 2019). Additionally, *Burkholderia* from the rhizosphere of *Baphicacanthus cusia*, as well as *Pseudomonas* and *Pantoea* strains from the rhizosphere of *Salvia miltiorrhiza*, can also directly participate in indigo biosynthesis and phenolic compound accumulation (Zeng et al., 2018; You et al., 2017).

Rhizosphere fungi are another important group within rhizosphere microorganisms, and their diversity plays a crucial role in stabilizing soil ecosystems, promoting plant growth, and directly participating in nutrient cycling between soil and plants, thereby making significant contributions to soil ecology (Adedayo and Babalola, 2013). Functionally, they are categorized into saprophytic fungi and symbiotic mycorrhizal fungi: beneficial fungi can enhance plant root activity, promote growth, and improve disease resistance, whereas harmful fungi may induce plant diseases or even lead to plant mortality (Finzi Razavi et al., 2016; Li Destri Nicosia et al., 2015). Among them, arbuscular mycorrhizal (AM) fungi form symbiotic associations with most terrestrial plants. By assisting hosts in absorbing water and mineral nutrients such as phosphorus (while acquiring organic nutrients from the hosts), they significantly influence plant secondary metabolism (Jiang et al., 2017; Welling et al., 2016; Kapoor et al., 2017). For instance, in *Salvia miltiorrhiza*, synthetic communities composed of fungi can significantly promote plant growth and improve the quality and yield of medicinal materials (Jia et al., 2024). Moreover, AM fungi are closely associated with the synthesis of phenolic compounds (Rosa-Mera et al., 2011). In addition, *Aspergillus flavus* can convert soluble arsenic into insoluble forms, reducing its toxicity to soil organisms to ensure plant growth (Mohd et al., 2019). In summary, the community diversity and functional specificity of rhizosphere microorganisms are the core research targets for elucidating the mechanisms underlying the growth regulation of medicinal plants and the accumulation of their active ingredients. Related research in this field has provided an important theoretical basis for the optimization of medicinal plant cultivation and the utilization of microbial resources.

This study investigated the relationship between rhizosphere microorganisms and *R. officinale* Baill. Using the second-generation NovaSeq platform for PE250 amplicon sequencing, we characterized bacterial and fungal communities in the 3-year-old plants. Our analysis focused on two key aspects: how these rhizosphere microorganisms influence soil physicochemical properties, and their impact on the accumulation of active components in medicinal plant *R. officinale* Baill.

## Materials and methods

### Sample collection

**Figure 1:**
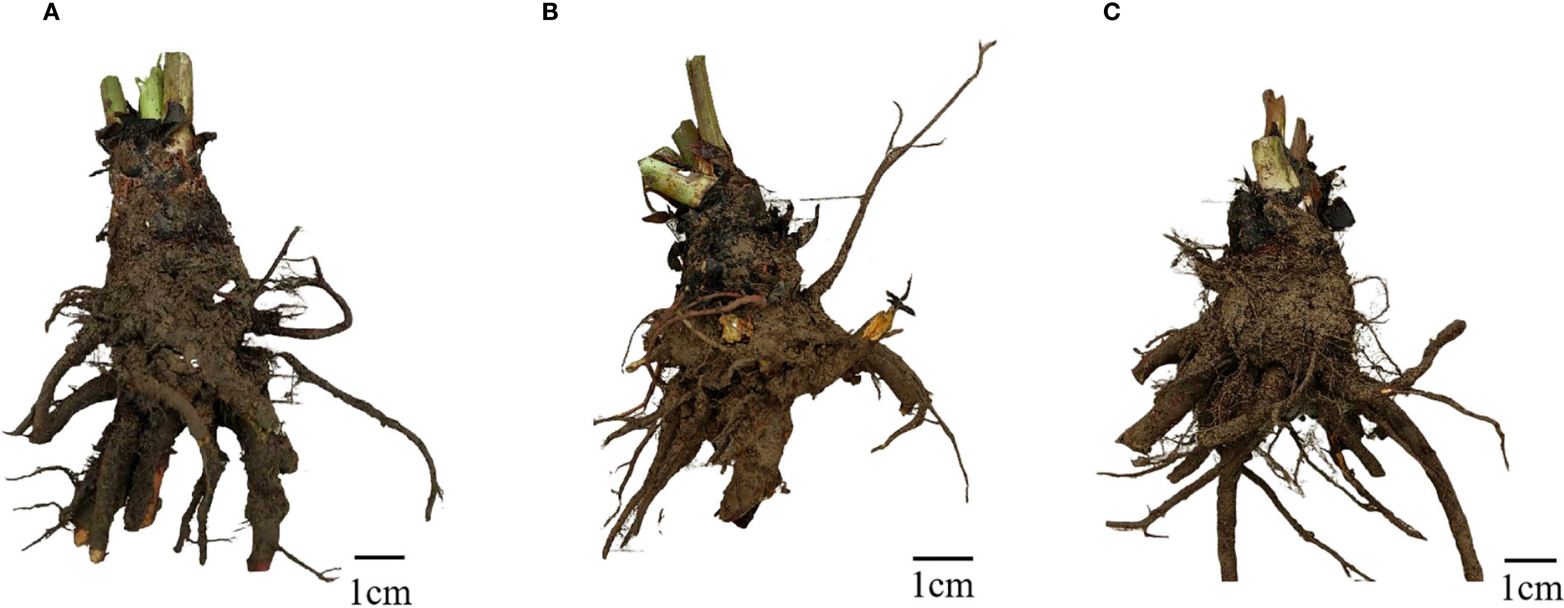
Sample photos of *R. officinale* Baill. from different origins. **(A)** ZB; **(B)** HB; **(C)** CQ.

Field sample was conducted in November 2023 across three major *Rheum officinale* producing regions: Shaanxi, Hubei and Chongqing. At each location, we collected three-year-old plants along with their rhizosphere soil (Li et al., 2021). Geographic coordinates for all sampling sites (longitude and latitude information) were recorded using Global Positioning System (GPS) (**Supplementary Table S1**). The study employed a replicated sampling design with three sampling points per region, each comprising three biological *R. officinale* Baill. replicates, yielding a total of nine sample batches. Notably, Zhenba County in Shaanxi Province served as a key sampling site due to its provincial recognition as a genuine producing area. All samples were collected from established medicinal cultivation bases. Three-year-old plants showing no visible signs of damage or disease were carefully selected (**Figure 1**, **Supplementary Figure S1**).

For soil sampling, the soil adhering to the roots of *R. officinal* was collected by gentle shaking, and rhizosphere soil samples tightly bound to the fibrous roots were carefully collected using sterilized brushes (**Supplementary Figure S2**). The frozen soil samples were dispatched to Shanghai Paisno Biotechnology Co., Ltd. for the detection of microbial diversity. Meanwhile, the remaining soil samples were air-dried to remove impurities, passed through a 20-mesh sieve, and then subjected to the determination of soil physical and chemical properties.

### Determination of 10 secondary metabolites in *R. officinale* Baill.

The high-performance liquid chromatography (HPLC) analysis was performed according to an established method for *R. officinale* Baill (Li et al., 2025), enabling simultaneous quantification of ten bioactive components: gcatechin, catechin, sennoside B, chrysophanol-8-*O*-glucoside, emodin-8-*O*-glucoside, aloe-emodin, rhein, emodin, chrysophanol and physcion. The fresh *R. officinale* Baill. herbs underwent the following steps. The workflow is summarized in the schematic below (Jiang et al., 2025), illustrating the key steps from sample preparation to chromatographic analysis (**Supplementary Figure S3**). First, the coarse outer skin was carefully scraped off. The peeled *R. officinale* Baill. was then sliced into 3.0 cm pieces. These slices were air-dried for seven consecutive days and subsequently dried in an electric blast drying oven at 50°C for seven days. Finally, the dried *R. officinale* Baill. slices were pulverized and sieved through a No. 4 sieve (with an inner diameter of sieve hole of 250 µm) to acquire samples appropriate for liquid phase detection. Precisely, 100 mg of each powdered sample was transferred to a 50 mL stoppered flask, mixed with 4.5 mL methanol, and weighed. After 30 minutes of ultrasonication (500W, 40kHz), samples were returned to room temperature, reweighted to compensate for solvent lose, then centrifuged at 11260 xg for 10 minutes. The supernatant was filtered through a 0.22 μm membrane. The reference substances were purchased from Chengdu Desite Biotechnology Co., Ltd. Individual reference standards were accurately weighed and dissolved in methanol to prepare10 mL stock solutions. A mixed working standard solution was prepared by combining 1.0 mL aliquots of each primary standard, followed by ten-fold dilution with methanol. All standard solutions were stored at 4 °C. Separation was achieved using a Maple Unitary C18 column (5 μm, 4.6 × 250 mm) with a binary mobile phase system: (A) methanol and (B) 0.2% phosphoric acid in water. The analysis was conducted at 30 °C with a flow rate of 1.0 mL/min, detection wavelength of 260 nm, and injection volume of 10 μL. Gradient elution was performed according to program **Supplementary Table S2**.

### Determination of soil physicochemical properties

Soil pH was measured with a Sardolus PB-10 pH meter. Total Nitrogen (TN) content was determined through sulfuric acid-catalyzed digestion. Flame photometric was used to quantify total Potassium (TK) content. For phosphorus analysis, total Phosphorus (TP) and available Phosphorus (AP) were determined using the NaOH fusion method followed by molybdenum-antimony anti-colorimetric method. Soil organic carbon and organic matter were determined via the potassium dichromate-concentrated sulfuric acid external heating method. Ammonium nitrogen (NH_4_^+^-N) and Nitrate nitrogen (NO_3_-N) was analyzed utilizing the potassium chloride extraction method. Soil Moisture Content (SWC) was determined gravimetrically by oven drying. Micronutrients analysis such as iron (Fe), manganese (Mn), zinc (Zn) and copper (Cu) was performed using acid extraction followed by atomic absorption spectrophotometry (Bao, 2000). All soil physical and chemical properties analyses were conducted by Yangling Xinhua Ecological Technology Co., Ltd.

### Soil DNA extraction, PCR amplification, Illumina sequencing, and data processing

Total soil DNA was extracted using the OMEGA Soil DNA Kit (D5635-02) (OMEGA Bio-tek, USA), with extraction quality verified by 0.8% agarose gel electrophoresis. For bacterial community analysis, the V3-V4 hypervariable region of 16S rRNA gene was amplified using universal primers 338F: (5’-ACTCCTACGGGAGGCAGCAG-3’) and 806R: (5’-GGACTACHVGGGTWTCTAAT-3’), with diluted DNA as template. Fungal community analysis targeted the ITS1 region using primers ITS1F(5’-GGAAGTAAAAGTCGTAACAAGG-3’) and ITS2R(5’-GCTGCGTTCTTCATCGATGC-3’) (Shanghai Paisano Biotechnology Co., Ltd., Shanghai). PCR products were purified using the Axygen Gel Recovery Kit (OMEGA, USA), followed by library preparation with the TruSeq Nano DNA LT Library Prep Kit (Illumina). Paired-end (2 × 250 bp) sequencing was performed on the Illumina MiSeq platform (Illumina, San Diego, CA) at Shanghai Personalbio Technology Co., Ltd.

### Statistical analysis of data

The paired-end sequencing was conducted using the NovaSeq 6000 SP Reagent Kit (500 cycles) (Illumina Shanghai Personalbio Technology Co., Ltd., Shanghai). Raw sequence data were demultiplexed using the demux plugin, followed by primer removal with cutadapt plugin. Subsequent processing included quality filtering, denoising, read merged and chimera removal using DADA2 plugin to generate amplicon sequence variation (ASVs). The ASVs were using MAFFT, and phylogenetic trees with constructed with FastTree2. Bioinformatic analyses were primarily performed in QIIME2 and R (Bokulich et al., 2018). Alpha diversity indices (Chao1 index and Shannon index) were calculated from the ASV table and visualized as boxplots. Microbial taxonomic composition and relative abundance were analyzed using MEGAN and GraPhlAn (Asnicar et al., 2015; Huson et al., 2011). Beta diversity was assessed through principal coordinates analysis (PCoA) based on the Bray-curtis dissimilarity matrices, with community composition differences visualized in two-dimensional sorting plots. Statistical analyses were conducted by using SPSS26.0 for one-way ANOVA, Kruskal-Wallis and Duncan’s multiple comparison test (α=0.05). GraphPad Prism 8 software was used for plotting.

## Results

### The content of bioactive ingredients in *R. officinale* Baill. exhibits interregional variations

**Figure 2:**
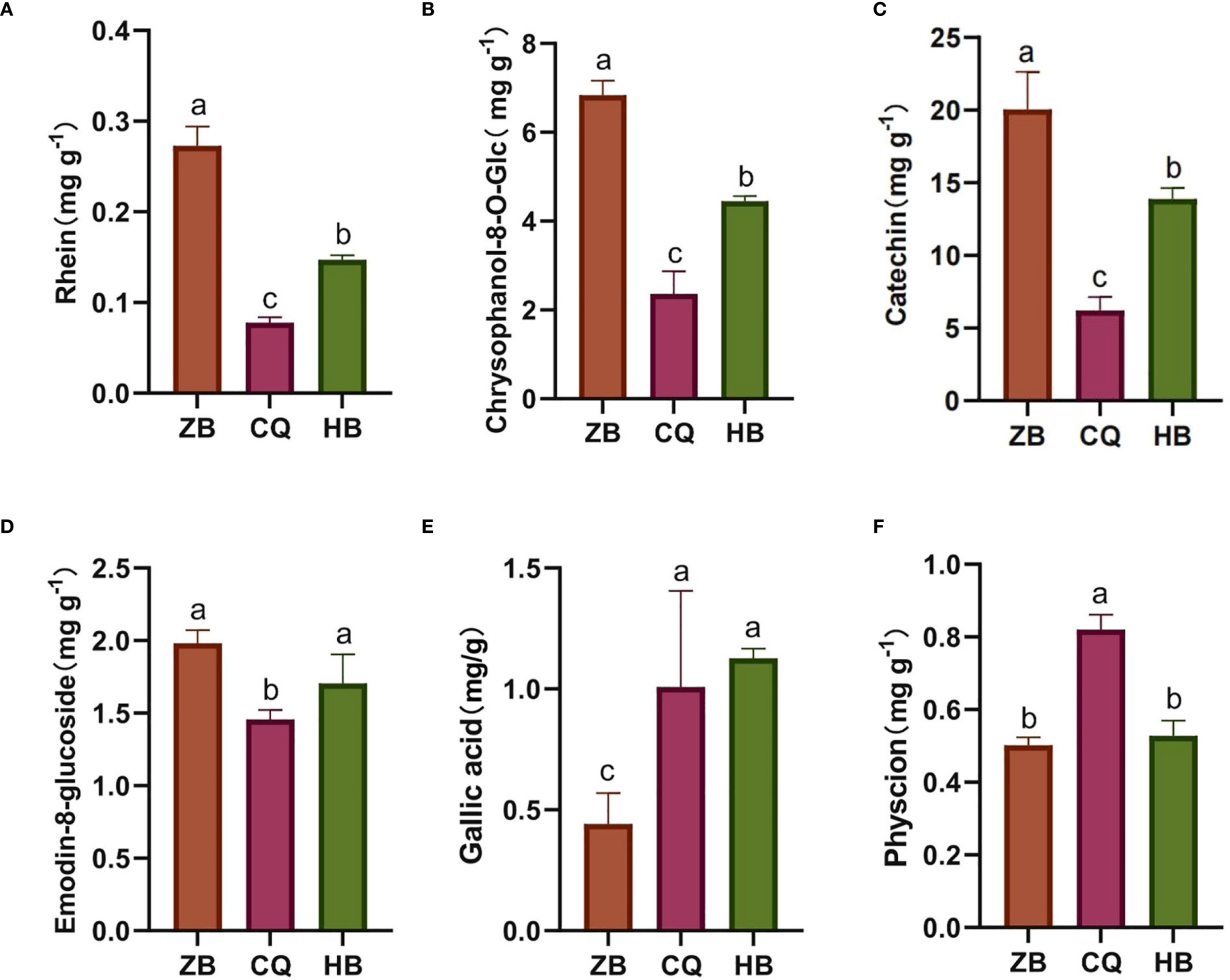
**(A-F)** is the numbering of the active ingredients in *R. officinale* Baill. The content of 6 effective components in *R. officinale* Baill. from 3 origins. The abscissa is the sample grouping, and the ordinate is the content of the components. Data are the mean of three replicates ± SE (standard error); different letters indicate significant differences at p < 0.05 according to analysis of variance (ANOVA).

To investigate the phytochemical variability of medicinal *R. officinale* Baill. from different geographical origins, we quantitatively analyzed ten characteristic bioactive ingredients using HPLC (**Supplementary Figure S4**). Comparative analysis revealed significant compositional variations in six components including rhein (**Figure 2A**), chrysophanol-8-*O*-glucoside (**Figure 2B**), catechin (**Figure 2C**), emodin-8-*O*-glucoside (**Figure 2D**), gallic acid (**Figure 2E**) and physcion (**Figure 2F**), whereas the remaining four components exhibited relatively Table Sconcentrations without statistically significant difference (**Supplementary Figure S5**). Specifically, *R. officinale* Baill. from ZB exhibited significantly higher levels of rhein (3.50 × CQ, 1.85 × HB), chrysophanol-8-*O*-glucoside (2.89 × CQ, 1.54 × HB), catechin (3.30 × CQ, 1.48 × HB) and emodin-8-*O*-glucoside (1.36 × CQ, 1.61 × HB) but lower gallic acid content compared to other regions. Conversely, CQ showed the highest physcion content (1.63 × ZB, 1.56 × HB). No significant interregional differences were observed for sennoside B, emodin, aloe-emodin, or chrysophanol. These findings demonstrate distinct chemotypic profiles associated with geographical origins, which may influence the pharmacological properties of *R. officinale* Baill. products.

### Analysis of the composition and diversity of fungal and bacterial colonies in rhizosphere soil

To investigate the potential correlation between rhizosphere microbial communities and phytochemical variations in *R. officinale* Baill., we performed comprehensive analysis of bacterial and fungal populations in the rhizosphere soils from different cultivation regions. Microbial community analysis was performed on nine rhizosphere soil samples collected from three geographic regions using Illumina HiSeq high-throughput sequencing. The rarefaction curves of ITS (**Supplementary Figure S6A**) and 16S rRNA (**Supplementary Figure S6B**) sequencing demonstrated the continuous emergence of novel operational taxonomic units (OTUs) with increasing sequencing effort, indicating previously uncharacterized microbial diversity. Curve plateauing at sufficient sequencing depth, suggesting adequate coverage of extant microbial diversity with samples (Good’s coverage >99%). For fungi, the sequencing depth ranges from 0 to 60,000 reads, and the effective sequencing depth (saturation depth) is 10,000–60,000 reads. For bacteria, the sequencing depth ranges from 0 to 25,000 reads, with a saturation depth of 5,000–10,000 reads. The asymptotic behavior of the curves confirms that the sequencing depth achieved was sufficient to capture the majority of bacterial and fungal diversity present in the samples, while further sequencing would primarily reveal rare taxa.

**Figure 3:**
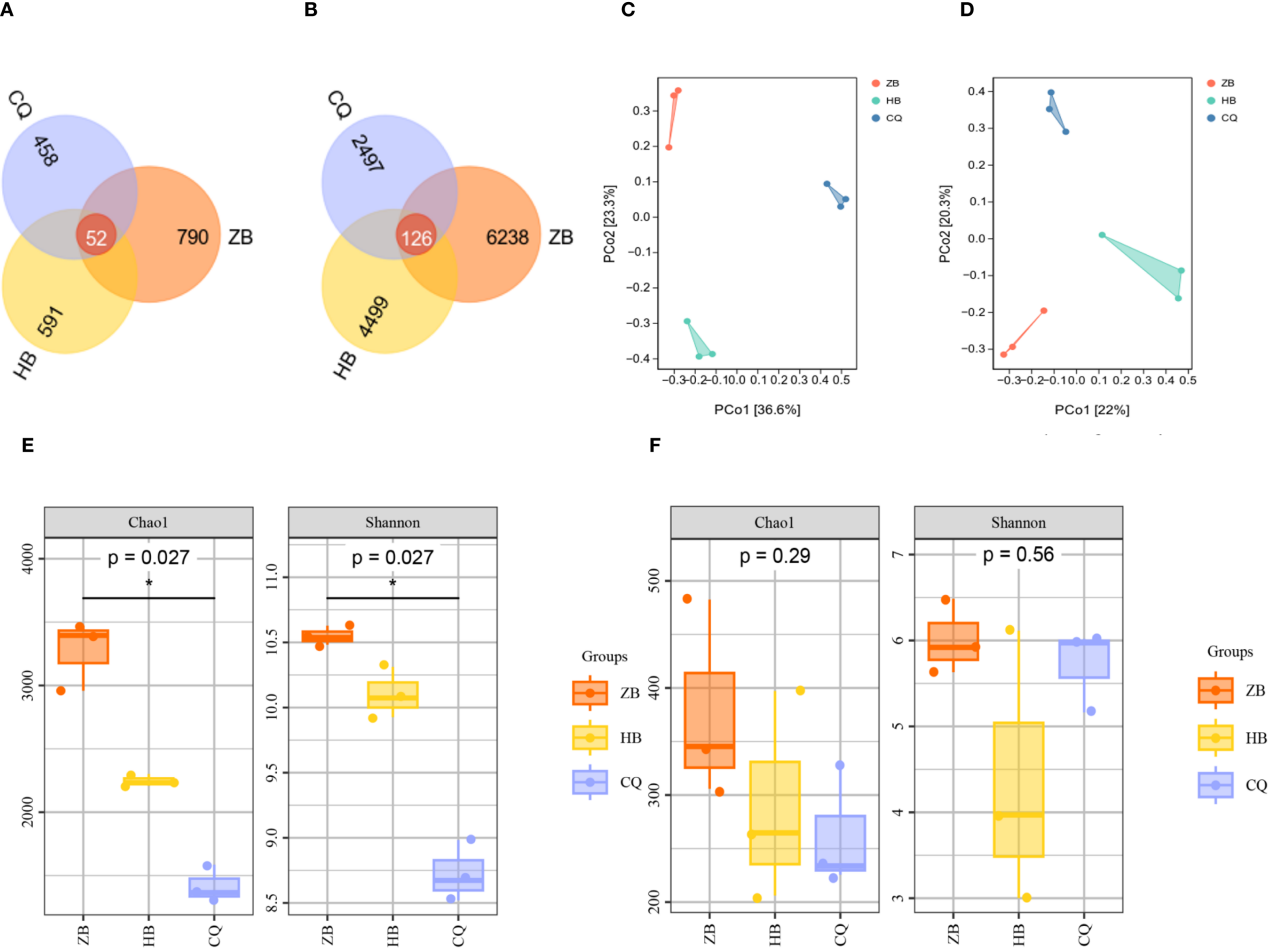
Venn diagrams depicting fungi **(A)** and bacteria **(B)** in the rhizosphere soil of *R. officinale* Baill. across three regions. PCoA analysis of fungi **(C)** and bacteria **(D)** in rhizosphere soil of *R. officinale* Baill. among three regions. α diversity analysis of bacteria **(E)** and fungi **(F)** in the rhizosphere soil of *R. officinale* Baill. in three regions. The Venn diagram shows the number of OTUs common and unique to the three regions, with different colors representing different regions. Each point in the PCoA diagram represents a sample, and the dots of different colors indicate different groupings. The percentage in the coordinate axis bracket represents the proportion of the sample difference data (distance matrix) that the corresponding coordinate axis can interpret. Box plots visually compare diversity between groups. α diversity index reflects the species richness and uniformity in the sample. Higher index values indicate higher microbial community complexity.

Microbial community analysis was conducted at 97% sequence similarity threshold to determine operational OTUs in rhizosphere soil of *R. officinale* Baill. from three regions. Venn diagram analysis revealed distinct distribution patterns of shared and unique OTUs. For the fungal community (**Figure 3A**), there were a total of 1891 OTUs in the three regions, and only 52 OTUs were shared among all three regions, ZB region exhibited the highest diversity with 842 total OTUs, accounting for 44.53% of the total. While, HB region contained 643 total OTUs, accounting for 34.00%. And CQ region showed 616 total OTUs, accounting for 26.97%. For the bacterial community, a total of 13,360 OTUs were present in the three regions, of which 126 OTUs were common to all regions (**Figure 3B**). The samples from ZB demonstrated maximum diversity, with a total of 6364 OTUs, accounting for 47.63% of the total. Whereas, samples form HB contained 4625 OTUs, accounting for 34.62%, and samples from CQ showed only 2623 OTUs, accounting for 13.63%. The overall microbial diversity followed a consistent regional pattern: ZB>HB>CQ (p<0.05), indicating significantly higher microbial diversity in ZB rhizosphere soils compared to other regions.

Alpha diversity analysis was performed to assess microbial community characteristics across different regions. The Chao1 index, which estimates species richness, showed higher values corresponding to greater community richness. The Shannon index, incorporating both species richness and evenness, indicated higher community diversity with increasing values. Both indices followed the same trend: ZB (fungi: Chao1 = 377.92, Shannon=6.01; bacteria: Chao1 = 3274.80, Shannon=10.55) > HB (fungi: Chao1 = 289.33, Shannon=4.36; bacteria: Chao1 = 2247.59, Shannon=10.10) > CQ (fungi: Chao1 = 262.11, Shannon=5.72; bacteria: Chao1 = 1419.09, Shannon=8.73). According to bacterial communities, significant regional variations were detected in both Chao1(P<0.05) and Shannon indices (P<0.05) (**Figure 3C**). Chao1 index ranking: ZB>CQ>HB. Shannon index pattern: CQ>HB>ZB. Regarding fungal communities, no statistically significant differences were detected in either Chao1 (P >0.05) or Shannon (P >0.05) (**Figure 3D**) indices among regions. These results demonstrate distinct spatial patterns in microbial diversity, with fungal communities showing consistent richness-diversity.

Principal Component Analysis (PCA) was employed to assess beta diversity patterns among sampling regions. For fungal communities (**Figure 3E**), the first two principal coordinates (PCo1 and PCo2) explained 42.03% of total variation (PCo1:22%; PCo2 20.3%). Bacterial communities (**Figure 3F**) showed stronger separation, with PCo1 (36.6%) and PCo2 (23.3%) collectively accounting for 59.9% of observed variation. This indicates that the selected principal components are effective in capturing the major patterns of difference in the raw data. In the PCoA diagram, the soil samples of ZB, HB and CQ were distributed in different quadrants and were far away from each other, indicating that there was significant spatial heterogeneity in the composition of fungal and bacterial communities in rhizosphere soil in the three regions.

### Composition of fungal and bacterial communities

**Figure 4:**
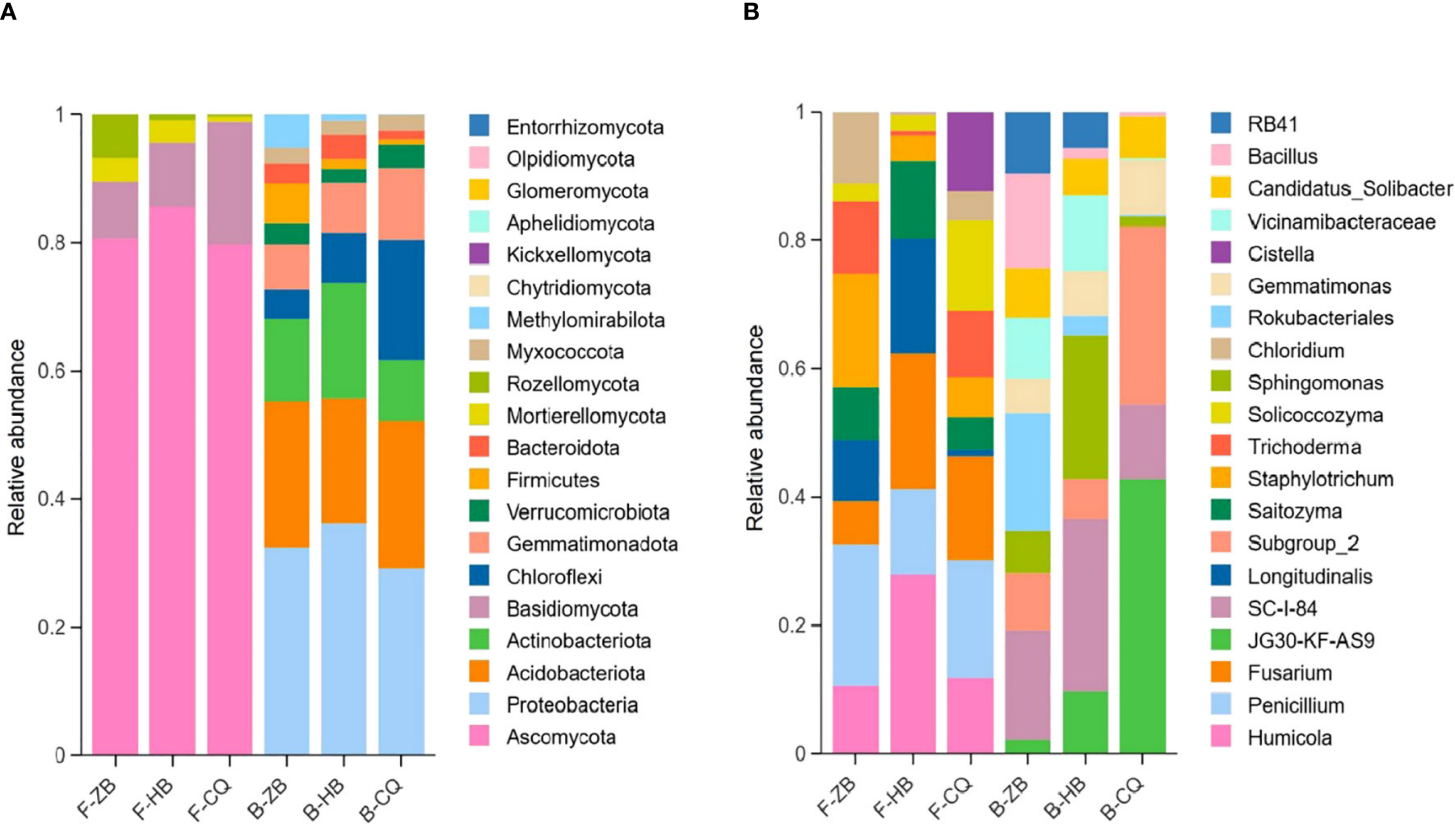
Relative abundance of fungi at the phylum **(A)** and genus **(B)** levels in the cultivated soil of *R. officinale* Baill. from three origins. F, fungi; B, bacteria. The picture shows the top 10 taxa by relative abundance at the phylum and genus level. The colors distinguish between microbial taxa, while the x-axis indicates the sample name and the y-axis indicates the relative abundance (%).

We further analyzed bacterial and fungal communities in *R. officinale* Baill. rhizosphere soils in Baill. three regions. Microbial abundance data were normalized during analysis, and species were matched in the NCBI database. Based on the horizontal abundance data of soil fungi in ZB, HB and CQ, the predominant fungal phylum across all regions was *Ascomycota*, accounting for 73.08%-92.14% of total fungal sequences. Secondary phyla included *Basidiomycota* (2.27%-15.30%), *Rozellomycota* (0.22%-1.52%) and *Mortierellomycota* (0.33%-3.79%), while other phyla each represented was less than 0.1% (**Figure 4A**). This consistent dominance pattern suggests *Ascomycota*’s ecological adaptation to the studied rhizosphere environments. However, in accordance with the bacterial community structure, the regional variations in the composition bacterial phyla were more pronounced. The horizontal abundance distribution of soil bacteria phyla in the three regions was as follows: *Proteobacteria* (30.20%) and *Acidobacteria* (20.30%) were the dominant phyla in ZB samples, followed by *Actinobacteriota* (11.71%), *Chloroflexi* (4.41%), *Verrucomicrobiota* (3.26%), *Bacteroidota* (2.97%) and *Gemmatimonadota* (6.22%). *Proteobacteria* (37.39%) and *Actinobacteriota* (18.11%) were the dominant categories in HB samples, followed by *Acidobacteria* (16.11%), *Chloroflexi* (5.72%), *Gemmatimonadota* (5.98%), *Verrucomicrobiota* (2.23%) and *Bacteroidota* (5.23%). *Proteobacteria* (26.77%) and *Acidobacteriota* (21.27%) were the dominant phyla in the CQ sample, followed by *Gemmatimonadota* (9.31%), *Verrucomicrobiota* (3.01%), *Chloroflexi* (19.69%), *Bacteroidota* (1.08%) and *Myxococcota* (1.74%) (**Figure 4A**). The microbial communities were predominantly composed of known beneficial taxa, including plant-growth promoting *Proteobacteria* and organic matter-degrading *Acidobacteria* in bacteria, along with *Ascomycota* in fungi. The differential abundance patterns suggest region-specific environmental selection pressures shaping microbial community assembly.

The top 10 soil fungal genus in ZB, HB and CQ were *Penicillium*, *Humicola*, *Fusarium*, *Longitudinalis*, *Staphylotrichum*, *Varicosporellopsis*, *Solicoccozyma*, *Oidiodendron*, *Saitozyma* and *Trichoderma* (**Figure 4B**). Among them, *Penicillium* (5.59%) was the most abundant in ZB, *Humicola* (41.13%) was the most abundant in HB, and *Penicillium* (10.75%) was the most abundant in CQ. Notably, *Fusarum* showed significant regional variation (CQ:6.40% > HB:2.86 > ZB: 2.11%). Eight of the den dominant fungal genera are known beneficial microorganisms, with *Trichoderma* and *Penicillium* being particularly notable for their plant-growth promoting properties. The top 10 species of soil bacteria were *JG30-KF-AS9*, *SC-I-84*, *Sphingomonas*, *Subgroup_2*, *Rokubacteriales*, *Vicinamibacteraceae*, *Gemmatimonas*, *RB41*, *KD4–96* and *Candidatus_Solibacter. Rokubacteriales* (4.84%) and *SC-I-84* (4.67%) were the dominant genera in ZB, *Sphingomonas* (6.87%) and *SC-I-84* (6.55%) were dominant in HB, and *JG30-KF-AS9* (14.56%) and *Subgroup_2* (7.29%) were dominant in CQ (**Figure 4B**). Ecologically significant beneficial genera including *Rokubacteriales*, *Sphingomonas* and *Gemmatimonas* showed higher relative abundance in ZB and HB (8.24%-8.89%) compared to CQ (2.72%). This distribution pattern potential functional differences in microbial-mediated soil processes across regions.

### Correspondence analysis on soil microorganisms from three different *R. officinale* Baill. cultivation regions

**Figure 5:**
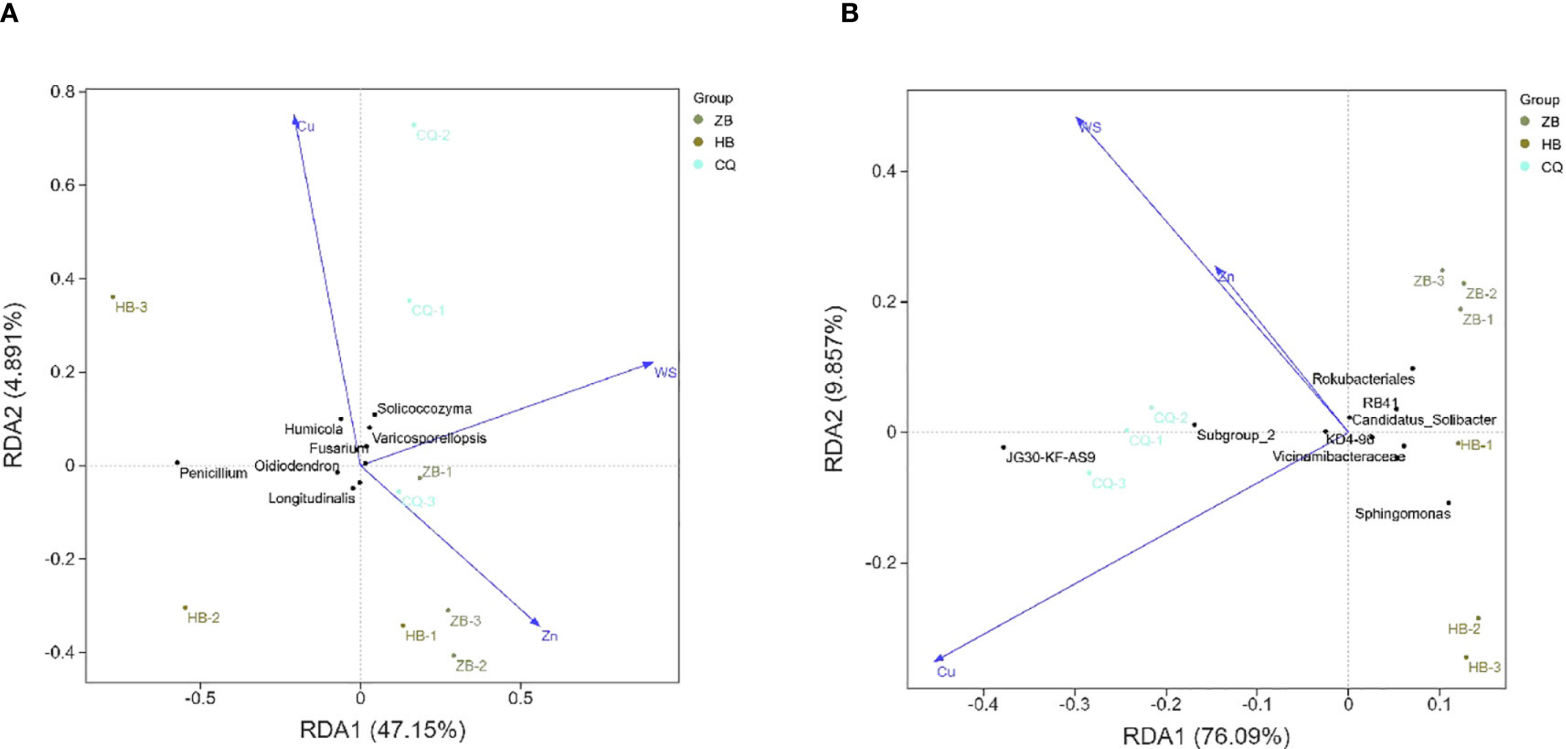
Redundancy analysis (RDA) of soil fungi **(A)** and bacteria **(B)** communities of *R. officinale* Baill. cultivated in three different locations and their environmental factors. The percentage of coordinate axes in the graph represents the degree of difference in the raw data it can interpret, with dots representing different samples and different colors representing different groupings. Arrows indicate environmental factors, and the length represents the degree to which the corresponding environmental factors are related to microorganisms. The angle between the arrows indicates the correlation between the two, with the acute angle positively correlated and the obtuse angle negatively correlated.

The soil samples from the three regions were roughly divided into three quadrants, indicating that there were some differences in the composition of fungal and bacterial communities among different soil samples. Among the fungal communities, the ZB region was dominated by *Penicillium* and *Humicola* (**Figure 5A**). *Penicillium* and *Humicola* were significantly enriched in the HB region. In the CQ area, *Solicoccozyma* and *Varicosporellopsis* are dominant. Among the physicochemical properties, the arrow of Cu was the longest and pointed to the negative direction of the RDA1 axis, indicating that it had the greatest impact on the community. The direction was consistent with the distribution of CQ samples, *Solicoccozyma* and *Varicosporellopsis*, indicating that Cu significantly affected the enrichment of CQ samples and *Solicoccozyma* and *Varicosporellopsis*. The WS pointed to the positive direction of the RDA2 axis, which was close to the CQ sample, which may have a certain effect on the fungal community in the CQ area. Zn points to the positive direction of the RDA1 axis, which is correlated with the distribution trend of the ZB group. In the bacterial community, the Cu arrow was the longest and pointed to the negative direction of the RDA1 axis, which was the most influential factor (**Figure 5B**). The direction was highly consistent with the distribution of CQ samples and *JG30*-*KF*-*AS9*, indicating that Cu may significantly affect the enrichment of CQ samples and *JG30*-*KF*-*AS9*. The arrows of Zn and WS are shorter and have a weaker influence. Zn points to the positive direction of the RDA1 axis, which is correlated with the distribution trend of samples in the ZB group. Taken together, the influencing factors of fungal and bacterial communities were about the same.

### Correlation analysis of soil microorganisms and bioactive components of *R. officinale* Baill.

**Figure 6:**
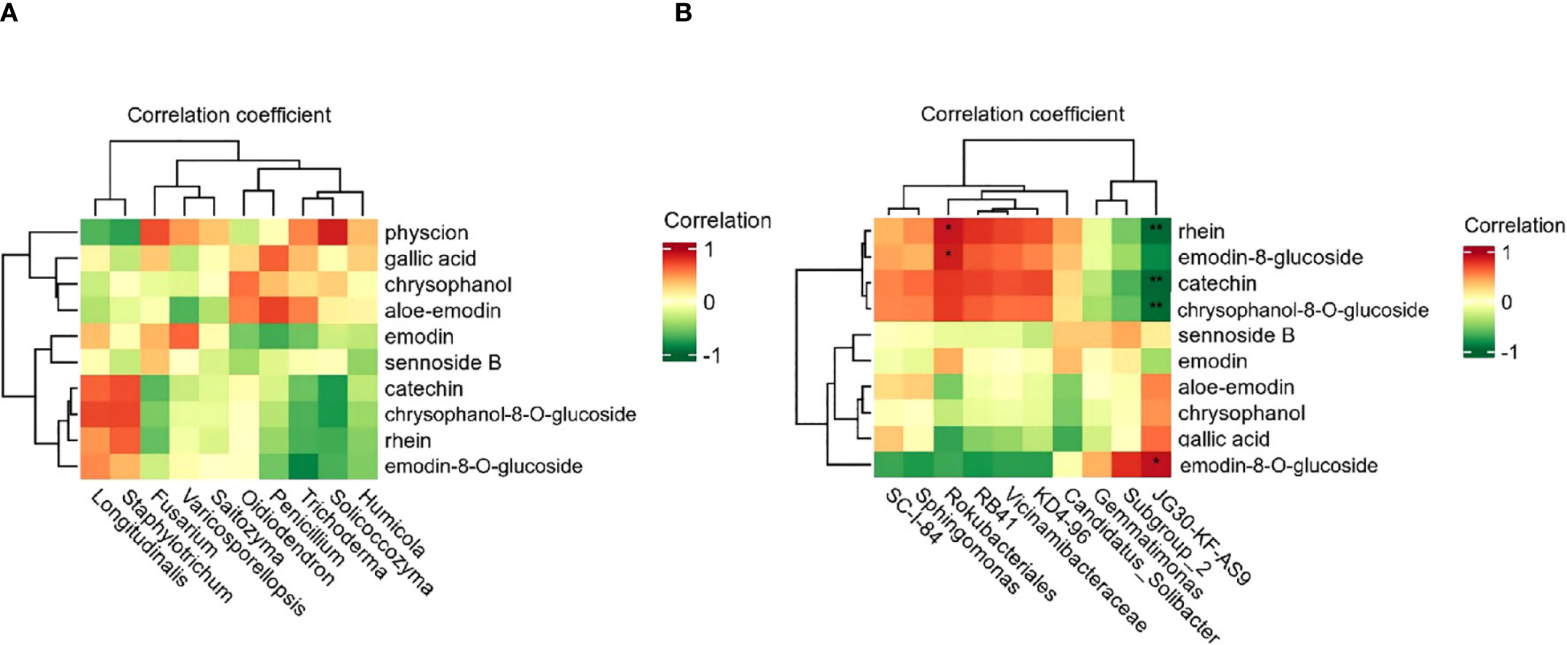
Correlation analysis between the content of active ingredients in *R. officinale* Baill. and the levels of fungi **(A)** and bacteria **(B)** in the cultivation soil. The abscissa in the figure represents the microbial species, and the ordinate is the ten active ingredients. The color of the square represents the level of the correlation coefficient.

Correlation analysis between soil factors and the active ingredient content in *R. officinale* Baill. was performed using SPSS software (version 27). It was performed between the top ten most abundant soil microorganisms (bacteria and fungi) and ten active ingredients in *R. officinale* Baill (Tohge, 2020). Firstly, the normality test of the data is carried out, and the Spearman correlation analysis method is selected to test the initial correlation coefficient and p-value according to the results. In order to control the risk of false positives caused by multiple comparisons, FDR correction was further used to process the p-value to more reliably identify the key soil factors affecting the formation of active ingredients in wolfberry. Fungal correlation showed that there were no fungi significantly associated with the ten active ingredients among the top 10 fungi in abundance (p>0.05) (**Figure 6A**). In the composition of the bacterial community, there was a significant correlation between two bacteria and the components (**Figure 6B**). *Rokubacteriales* was significantly positively correlated with rhein and emodin-8-*O*-glucoside (p<0.05), and *JG30-KF-AS9* was significantly negatively correlated with rhein, catechin, and chrysophanol-8-*O*-glucoside (p<0.01), and positively correlated with physcion (p<0.05).

### Differences in physicochemical properties of rhizosphere soil of *R. officinale* Baill. in different production areas

To explore whether the differences in soil are related to the variations in the components of *R. officinale* Baill., we conducted test on various physical and chemical indicators of the soil.

Significant regional variations in soil physicochemical properties were observed among the three study areas (**Table 1**). Soil pH analysis revealed neutral conditions in ZB (pH 6.5-7.5), while HB and CQ exhibited acidic soils (pH 4.5-5.5). Trace element analysis demonstrated distinct distributions patterns: iron (Fe) concentrations were markedly elevated in HB (6.78 × ZB, 1.38 × CQ; p<0.01), whereas ZB showed the highest manganese (Mn) levels (5.91× HB, 1.20× CQ; p<0.01). CQ soils contained significantly greater Copper (Cu) (2.01 × ZB, 1.24 × HB; p<0.05), while Zinc (Zn) showed no significant interregional differences. Nutrient analysis revealed HB’s superior fertility, with significantly higher concentrations of total nitrogen (TN: 1.65 × ZB, 1.95 × CQ), organic matter (OM: 2.04 × ZB, 2.48 × CQ), available phosphorus (AP: 7.74 × ZB, 8.46 × CQ), and total phosphorus (TP: 2.57 × ZB, 1.92 × CQ; all p<0.01). CQ exhibited the highest total potassium (TK: 1.45 × ZB, 1.12 × HB; p<0.05) and ammonium nitrogen (NH_4+_-N: 6.48 × ZB, 1.53 × HB; p<0.01). ZB showed predominant nitrate nitrogen (NO_3_^-^-N: 2.93 × HB, 8.01 × CQ; p<0.01), while water-soluble content (SWC) was significantly lower in HB compared to other region.

**Table 1:** Soil physicochemical properties.

Index	ZB	HB	CQ
pH	6.1333 ± 0.0907	5.13 ± 0.01	4.82 ± 0.0529
Zn	2.3091 ± 0.1865	2.2122 ± 0.0538	2.3133 ± 0.0578
Fe	14.1382 ± 1.3556	95.927 ± 3.7401	69.6704 ± 2.978
Mn	62.2394 ± 2.8578	10.5285 ± 0.2194	51.728 ± 0.8646
Cu	0.9123 ± 0.073	1.4753 ± 0.0413	1.8363 ± 0.068
TN	1.7973 ± 0.0491	2.9649 ± 0.0966	1.5225 ± 0.0144
OM	29.6375 ± 0.4334	60.4537 ± 0.6611	24.335 ± 0.3768
AP	23.0333 ± 0.9793	178.1667 ± 5.8126	21.0667 ± 0.3253
TP	0.5906 ± 0.0121	1.5155 ± 0.0147	0.7906 ± 0.0076
TK	15.4504 ± 0.4246	20.0417 ± 0.1015	22.4769 ± 0.3172
AK	241.3333 ± 6.4291	260.5581 ± 3.8116	147.3333 ± 6.6583
NH_4_^+^-N	3.9781 ± 0.411	16.8209 ± 0.5874	25.783 ± 0.9739
NO_3_^-^-N	36.2577 ± 1.8023	12.3743 ± 1.0447	4.5264 ± 0.4299
SWC	33.228 ± 1.5133	28.1955 ± 0.3253	33.3004 ± 0.7698

### Correlation analysis of soil physical and chemical properties and bioactive components of *R. officinale* Baill.

**Figure 7:**
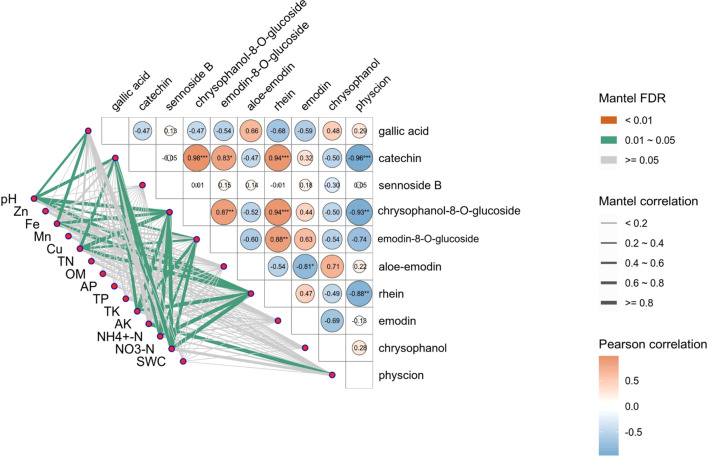
Correlation analysis between the content of active ingredients in *R. officinale* Baill. and soil properties. The lower left line represents the Mantel test results of soil physical and chemical properties and active ingredients. The thickness of the line represents the overall correlation coefficient, and the color of the line represents the significance test result of the correlation coefficient. The triangle is a heat map of correlations between substances, with cell color blocks indicating the size of the correlation coefficient, red indicating positive correlation, and blue indicating negative correlation.

The correlation between soil properties and the active ingredients of *R. officinale* Baill. was further discussed, and the p-value was FDR corrected (**Figure 7**; **Supplementary Table S3**). The results showed that Zn, Mn, TN, OM, AP, TP and SWC were not significantly correlated with the 10 active ingredients. Specifically, pH was significantly positively correlated with gallic acid, catechin, chrysophanol-8-*O*-glucoside, and rhein (P <0.05); Fe and gallic acid, chrysophanol-8-*O*-glucoside and rhein were significantly positively correlated. Cu, NH_4_^+^-N and NO_3_-N were significantly positively correlated with catechin, chrysophanol-8-*O*-glucoside, emodin-8-*O*-glucoside and rhein. TK and catechin, chrysophanol-8-*O*-glucoside, emodin-8-*O*-glucoside and rhein were significantly positively correlated. AK was significantly positively correlated with physcion.

## Discussion

A growing body of research has shown that *R. officinale* Baill. has a variety of pharmacological properties, including anti-inflammatory, anticancer, antibacterial, and antiviral, and can also be used as an expectorant. It has been recognized as effective in treating a variety of conditions, including stomach pain, nausea, and vomiting (Keshavarzi et al., 2021). The medicinal properties of *R. officinale* Baill. stem from the synergistic effects of several active ingredients (**Supplementary Table S4**). Gallic acid and catechin belong to tannins, sennoside B belongs to dianthranone, chrysophanol-8-*O*-glucoside and emodin-8-*O*-glucoside belong to conjugated anthraquinone, and aloe-emodin, rhein, emodin, chrysophanol and physcion belong to free anthraquinone. According to the results of HPLC analysis, there exist distinct regional patterns in bioactive compound accumulation. Specifically, ZB region exhibited the highest concentrations of tannins (gallic acid, catechin), dianthrones (sennoside B), and conjugated anthraquinones (chrysophanol-8-*O*-glucoside, emodin-8-*O*-glucoside). Conversely, free anthraquinone (aloe-emodin, rhein, emodin, chrysophanol, physcion) were minimally abundant in ZB but reached peak levels in CQ region. Studies have revealed that the pharmacological effect of tannins predominantly manifest as astringency and antidiarrheal properties (Xu et al., 2024). Regarding anthone, its pharmacological effects mainly include laxative and promoting of bile secretion. When combined with anthraquinone, it exhibits a potent laxative effect. Moreover, it also possesses antioxidant functions. Free anthraquinone is associated with various effect such as antibacterial, anti-inflammatory, antitumor and antioxidant effects (Xiang et al., 2020). The pronounced inter-regional variability in active ingredients profiles underscore the significant impact of geographical factors on secondary metabolite biosynthesis in *R. officinale* Baill (Komatsu et al., 2006). These suggest that origin-specific quality variations may influence clinical efficacy.

Soil physicochemical properties serve as critical determinants of medicinal plant yield and quality through their influence on plant growth, nutrient uptake, secondary metabolism and microbial interaction (Lv G. et al., 2024). Through the association of soil physicochemical properties with active components, it was discovered that pH exhibited a strong correlation with the content of ten active components. In contrast, TN, AK, and heavy metal ions in soil were only correlated with specific individual components. Thus, we hypothesized that soil pH might be the core factor driving the quality variation of *R. officinale* Baill., consistent with observations in other medicinal species. Study revealed that soil stands as one of the crucial determinants of *Panax notoginseng* production, and soil physicochemical properties and soil microbiome have high contributions to the biomass and saponins of *Panax notoginseng* (Xia et al., 2024). Specific pH conditions proved beneficial for the synthesis and accumulation of THSG and total anthraquinones in *Polygonum multiflorum*, thereby enhancing the quality of the medicinal material (Wu et al., 2023). Enhanced medicinal quality of *Astragalus mongholicus* correlates with improved soil quality, particularly pH, soil organic matter (SOM), and calcium content (STCa). These factors directly regulate the biosynthesis of key bioactive compounds including asragalosides (I, II, and IV) and calycosin (Sun et al., 2020). Similar studies in *Lycium barbarum* found that soil pH mediates the accumulation of fruit flavonoids and total sugars, demonstrating both direct and indirect regulatory effects (Liu et al., 2022). It is worth noting that correlation analysis only provides preliminary insights into the potential association between soil physicochemical factors, active ingredients and microorganisms, but cannot establish a causal mechanism between the two, so the results need to be interpreted with caution.

Rhizosphere microorganisms can directly or indirectly regulate the accumulation process of plant active ingredients by building an “interaction network” with plant roots (Lv J. et al., 2024). In terms of nutrient uptake enhancement, nitrogen-fixing microorganisms can convert N_2_ in the air into ammonia nitrogen available to plants, and efficiently supplement nitrogen for plants. In addition, these microorganisms can synthesize plant hormones such as indoleacetic acid (IAA) to promote root growth, and cytokinins to enhance leaf photosynthesis and fruit development. At the same time, microorganisms can also regulate the physical and chemical properties of rhizosphere soil and create a suitable microenvironment for the accumulation of active ingredients (Hacquard et al., 2017; Zhong et al., 2022). Analysis of soil samples from different *R. officinale* Baill. cultivation regions revealed significant correlations between microbial community characteristics and specific environmental, with copper (Cu), zinc (Zn) and water content (WC) emerging as primary determinant. Heavy metal contamination, originating from mineral mining, fossil fuel combustion, and industrial/agricultural activities, has been documented to alter soil physicochemical properties and disrupt microbial composition and community structure through cadmium (Cd), arsenic (As), and Cu accumulation (Perveen et al., 2017). Experimental evidence demonstrates a concentration-dependent relationship between Cu exposure and microbial diversity reduction. Biolog analysis indicate an inverse correlation between CuCl_2_ concentration (0, 10, 20, 100 and 300 μmol/L) and Shannon diversity indices, confirming Cu’s suppressive effect on microbial diversity (Woźniak et al., 2019; Kong et al., 2006). In addition, it has been found that Cu has a particularly significant effect on the composition of rhizosphere bacterial communities, which may be due to the fact that Cu can not only destroy the cellular structure and function of microorganisms, but also change the spatial conformation of proteins by binding to specific proteins, produce harmful clumps, interfere with the functions of key proteins, lead to cellular metabolic disorders and signal transduction obstruction, enter a toxic stress state, and eventually die (An et al., 2023). In this study, Cu exhibited the strongest pronounced effects observed in the CQ region. This spatial pattern suggests localized Cu accumulation may drive significant microbiome alteration in CQ soils. In contrast, Zn and water content demonstrated comparatively weaker impacts on microbial communities. These results are only derived from high-throughput data analysis. The specific mechanisms still need to be further verified through experiments.

*Fusarium*, a prevalent pathogenic genus among the dominant fungi taxa, exhibits significant phytotoxicity toward medicinal plants. For example, in *Panax ginseng*, *Fusarium* can cause root rot, leading to root decay, which affects the ginseng’s absorption of water and nutrients, and in severe cases, the entire plant can die (Jun et al., 2022). When *Salvia miltiorrhiza* is infected by *Fusarium*, the content of effective components such as tanshinone will decrease significantly, thus affecting the medicinal value of Danshen (Yang et al., 2020). In medicinal plants like *Atractylodes macrocephala*, root rot and wilt diseases often occur, leading to poor growth, reduced yield, and diminished quality (Huang et al., 2021). Our data revealed a negative correlation between *Fusarium* and catechin levels, suggesting potential suppression of catechin biosynthesis, possibly through interference with phenylpropanoid pathway enzymes. This provides us with a clue that when *Fusarium* infects the plants, it may reduce the medicinal quality of *R. officinale* Baill. by directly or indirectly affecting the synthesis of catechin. Subsequently, molecular techniques can be combined with plant pathology methods for verification. As a beneficial fungus, *Trichoderma* can solubilize poorly soluble nutrients such as phosphorus and potassium in the soil by secreting organic acids, enzymes, and other substances. It converts these nutrients into forms that can be absorbed by plants, thereby enhancing the medicinal plants’ ability to absorb and utilize these nutrients and promoting plant growth and development. For instance, during the cultivation of *Astragalus membranaceus*, it facilitates plant growth by dissolving soil nutrients, promoting root elongation, and generating hormone-like substances (He et al., 2022). Correlation analysis revealed a modest positive association between *Trichoderma* and the concentration of physcion and aloe-emodin. This pattern suggests that *Trichoderma* might contribute to enhanced synthesis or accumulation of physcion and aloe-emodin. Conversely, *Trichoderma* exhibited significant negative correlations with emodin-8-*O*-glucoside, rhein, and chrysophanol-8-*O*-glucoside, indicating potential inhibitory effects on these metabolites. Regarding *Penicillium*, evidence suggests this fungal genus promotes growth and secondary metabolism in medicinal plants. For instance, mono-inoculation of the endophytic fungus *Penicillium steckii* in *Tripterygium wilfordii* significantly increased triptolide and tripterine production (Song et al., 2020). Based on this functional analogy, *Penicillium* may similarly facilitate aloe-emodin in *R. officinale* Baill. All correlation analyses were interpreted with careful consideration of inherent observational limitations. While these analyses reveal significant associations between microbial communities and metabolic profiles, they do not establish direct causation. To validate these findings and elucidate mechanistic relationships, we propose employing complementary experimental approaches, including microbial isolation of key operational taxonomic units, controlled co-cultivation systems integrating isolated microbes with plant tissues, and Table Sisotope-assisted metabolic tracing to quantify microbial contributions. This multi-approach will bridge the gap between correlative observations and causative inferences.

Notably, among the top ten bacteria phyla in relative abundance, many remain understudied regarding their functional roles. The bacteria taxa most strongly associated with bioactive components in *R. officinale* Baill. include *Rokubacteriales*, *JG30-KF-AS9*, *RB41*, *Subgroup_2* and. *RB41* belongs to the phylum *Acidobacteria*, which contributes significantly to soil carbon cycling. *Acidobacteria* generally account for approximately 20% of soil bacterial communities (Jones et al., 2009) and carry out ecologically beneficial functions including plant polymers degradation, iron cycling, and single-carbon compound metabolism (Pankratov et al., 2012; Lu et al., 2010). *RB41* and *Rokubacteriales* are significantly positively correlated with catechin, chrysophanol-8-*O*-glucoside, emodin-8-*O*-glucoside, and rhein, indicating that bacteria from these two genera may promote the accumulation of these four components. *Subgroup_2* and *JG30-KF-AS9* are extremely significantly positively correlated with physcion, suggesting that bacteria from these two genera may promote the synthesis and accumulation of physcion, while being negatively correlated to varying degrees with emodin-8-*O*-glucoside, rhein, catechin, and chrysophanol-8-*O*-glucoside, possibly inhibiting the synthesis and accumulation of these four components. Due to the limited sample size (N = 3), the statistical power of our analyses may be insufficient to detect subtle effects or interactions. Correlation analysis only provides preliminary insights into the potential association between soil physicochemical factors, active ingredients and microorganisms, but cannot establish a causal mechanism between the two, so the results need to be interpreted with caution.

While these correlation analysis provide preliminary insights into potential microbe-metabolite relationships in *R. officinale* Baill., their inherent limitations warrant cautious interpretation. Correlative data alone cannot establish causal mechanisms and may reflect indirect associations influenced by unmeasured environmental variables or microbial interactions. To validate the proposed roles of *RB41*, *Rokubacteriales*, *Subgroup_2*, and *JG30-KF-AS9* in metabolite modulation, targeted experimental approaches are recommended. For instance, *in vitro* co-culture assays with isolated bacterial strains and *R. officinale* Baill. tissues/cell lines to directly quantify effects on specific metabolite accumulation.

## Data Availability

The datasets presented in this study are publicly available. This data can be found here: https://www.ncbi.nlm.nih.gov, accession numbers PRJNA1288235 and PRJNA1320834.

## Supplementary material

Supplementary Figure 1 Schematic diagram of the sample preparation and HPLC analysis procedure.

Supplementary Figure 2 The content of four effective components in *R. officinale* Baill. sourced from three locations shows no obviously variations: sennoside B (A), emodin (B), aloe-emodin (C), chrysophano (D). Data are the mean of three replicates ± SE (standard error); different letters indicate significant differences at p < 0.05 according to analysis of variance (ANOVA). ZB for Zhenba, Shaanxi. CQ for Chongqing. HB for Hubei.

Supplementary Figure 3 Rhubarb plants from three medicinal rhubarb cultivation bases. A: ZB; B: HB; C: CQ.

Supplementary Figure 4 Soil sample for fungal ITS and bacterial 16S sequencing from three regions. ZB for Zhenba, Shaanxi. CQ for Chongqing. HB for Hubei.

Supplementary Figure 5 HPLC chromatograms of *R. officinale* Baill. from three production areas.

Supplementary Figure 6 Dilution curves of fungal ITS (A) and bacterial 16S (B) sequencing in soil samples. The abscissa is the flattening depth, and the ordinate is the alpha diversity index calculated 10 times.
