# Supplementary figures and images for: Comparative analysis of rhizosphere microbial communities and secondary metabolites in cultivated Rheum officinale from different regions of China
Source: Front Plant Sci. 2025 Sep 30;16:1650792. doi: 10.3389/fpls.2025.1650792 (PMC12518358; doi:10.3389/fpls.2025.1650792)

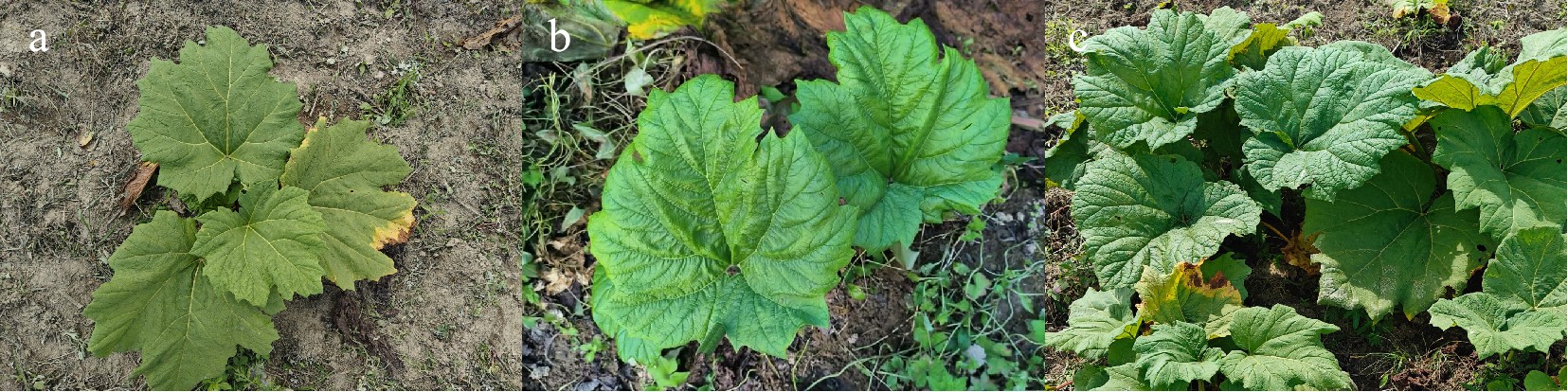

Supplement: Supplementary Figure 1 — Schematic diagram of the sample preparation and HPLC analysis procedure. [file Image1.jpg]

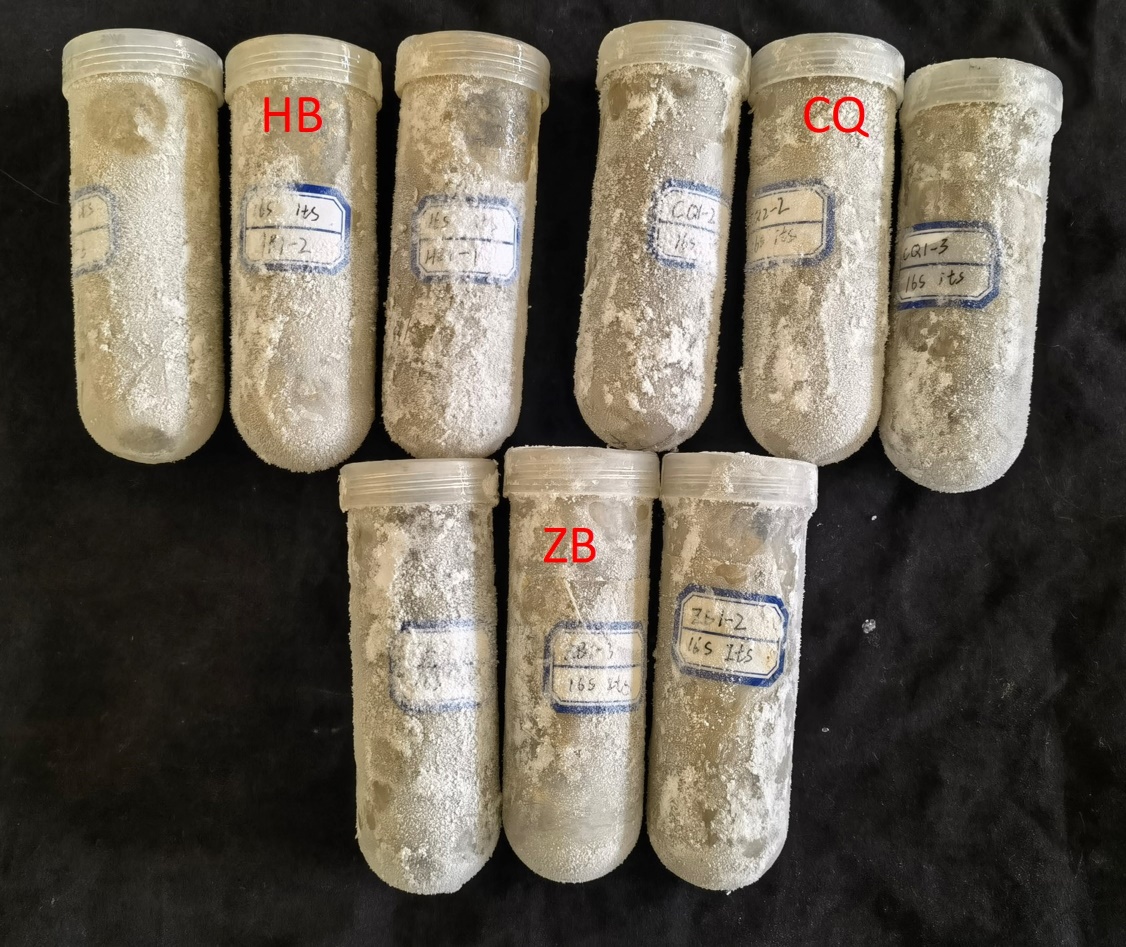

Supplement: Supplementary Figure 2 — The content of four effective components in R. officinale Baill. sourced from three locations shows no obviously variations: sennoside B (A), emodin (B), aloe-emodin (C), chrysophano (D). Data are the mean of three replicates ± SE (standard error); different letters indicate significant differences at p < 0.05 according to analysis of variance (ANOVA). ZB for Zhenba, Shaanxi. CQ for Chongqing. HB for Hubei. [file Image2.jpg]

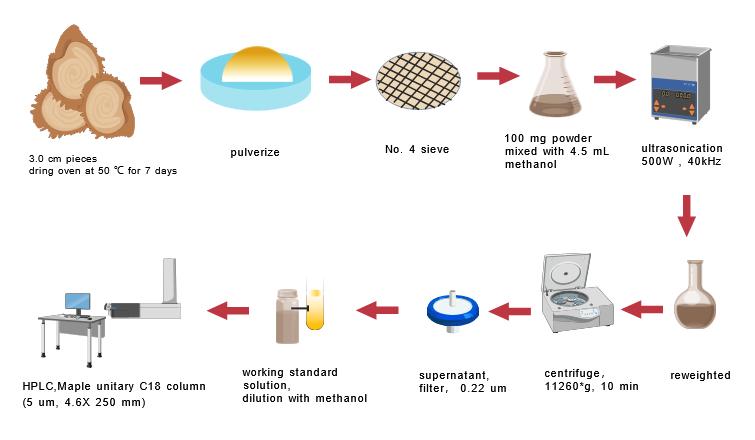

Supplement: Supplementary Figure 3 — Rhubarb plants from three medicinal rhubarb cultivation bases. A: ZB; B: HB; C: CQ. [file Image3.jpg]

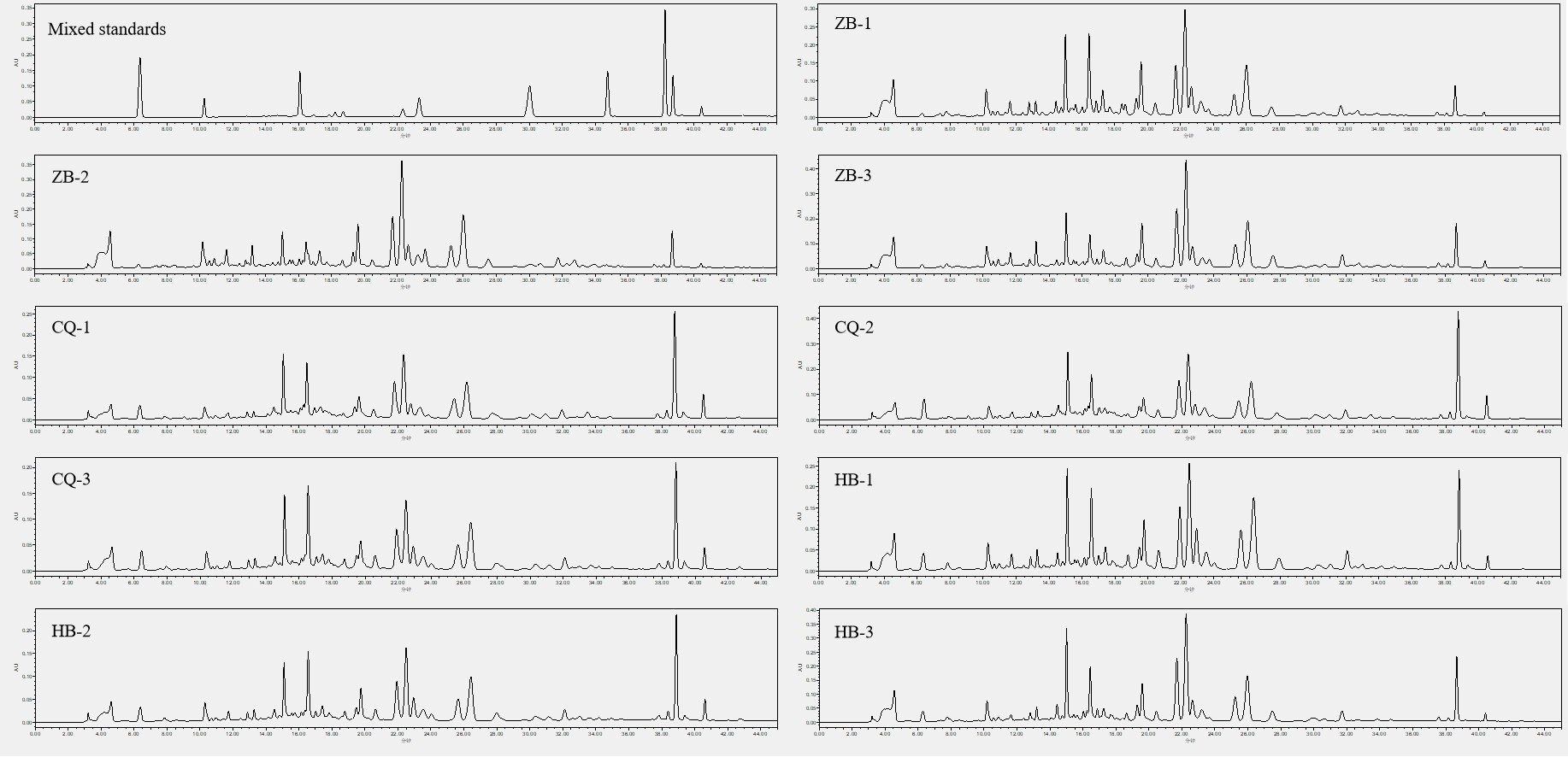

Supplement: Supplementary Figure 4 — Soil sample for fungal ITS and bacterial 16S sequencing from three regions. ZB for Zhenba, Shaanxi. CQ for Chongqing. HB for Hubei. [file Image4.jpg]

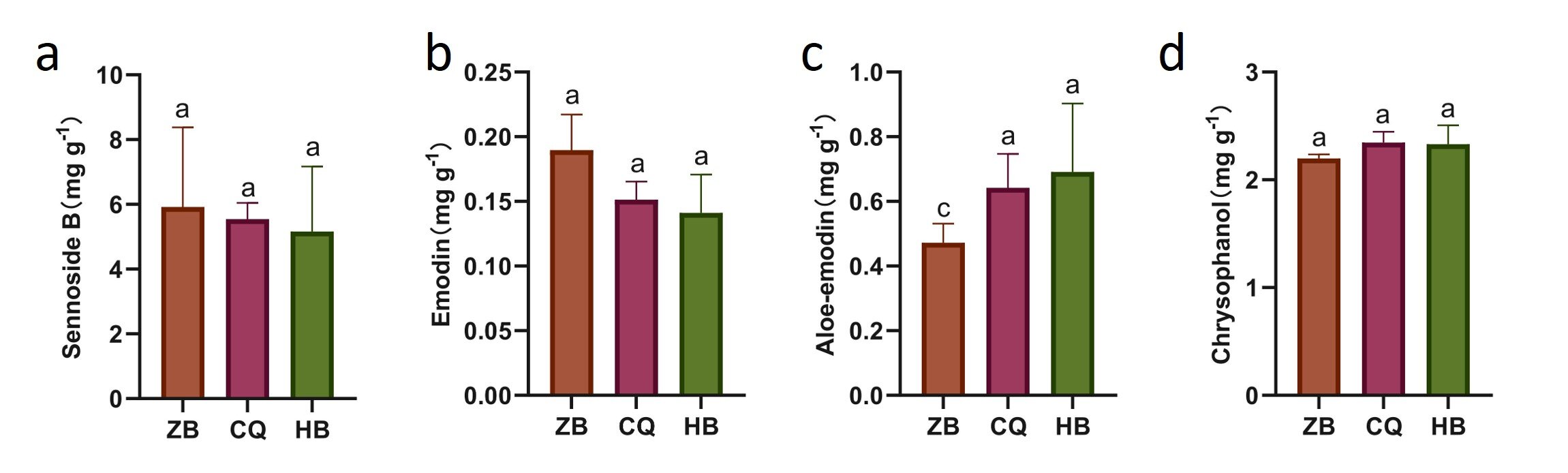

Supplement: Supplementary Figure 5 — HPLC chromatograms of R. officinale Baill. from three production areas. [file Image5.jpg]

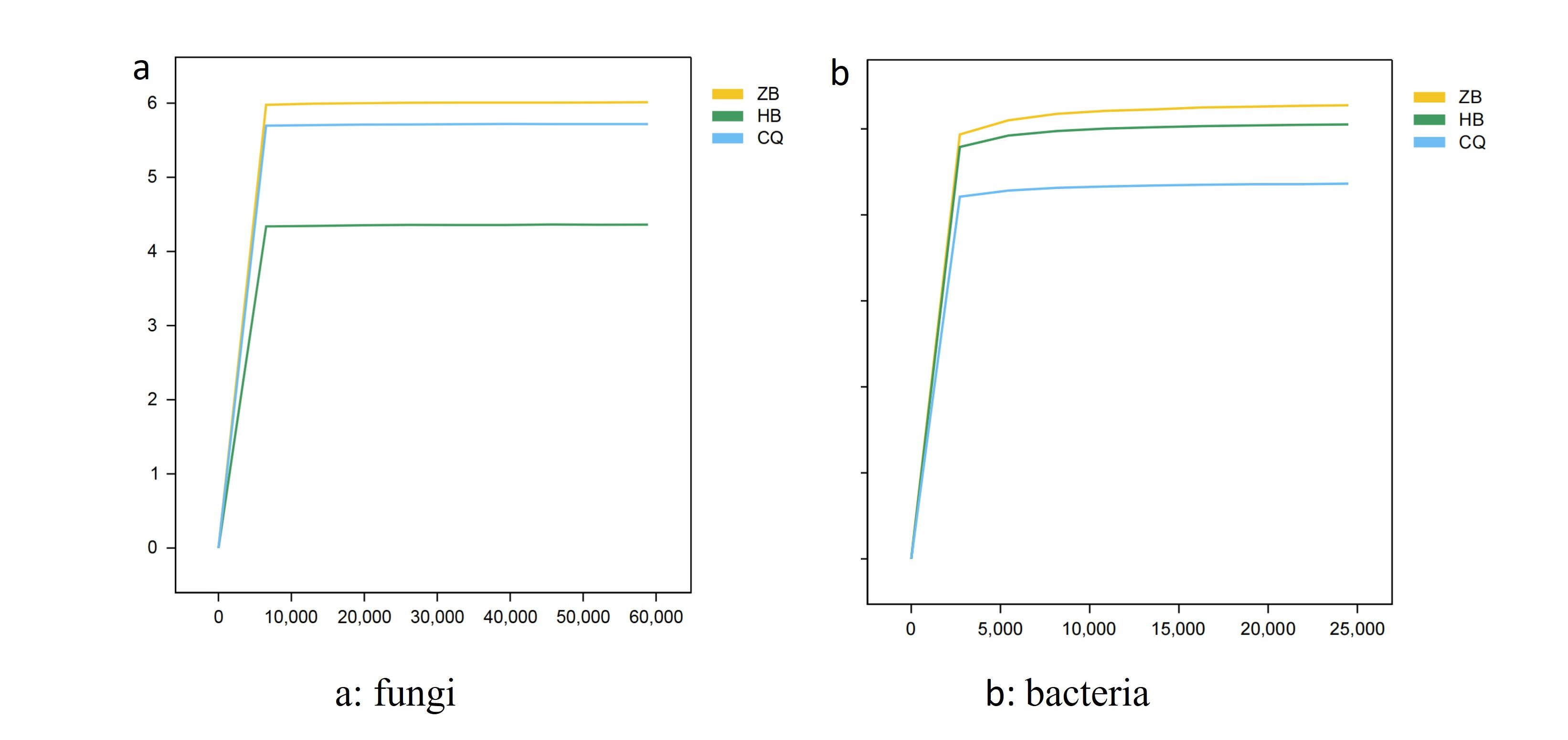

Supplement: Supplementary Figure 6 — Dilution curves of fungal ITS (A) and bacterial 16S (B) sequencing in soil samples. The abscissa is the flattening depth, and the ordinate is the alpha diversity index calculated 10 times. [file Image6.jpg]
