# Supplementary material for: Comparative analysis of rhizosphere microbial communities and secondary metabolites in cultivated Rheum officinale from different regions of China
Source: Front Plant Sci. 2025 Sep 30;16:1650792. doi: 10.3389/fpls.2025.1650792 (PMC12518358; doi:10.3389/fpls.2025.1650792)
Supplement: Supplementary file 7 [file Table1.docx]

Stable 1 Longitude, latitude and altitude information of three rhubarb plant samples and cultivated soil samples

| sample ID | Harvestingzonename | Longitude(°E) | Latitude(°N) | Avr.altitude(m) |
| --- | --- | --- | --- | --- |
| ZB | YuantanVillage,YanchangTown,ZhenbaCounty,ShaanxiProvince | 107°53′45″ | 32°25′22″ | 1290 |
| HB | YuanbaoTownship,LichuanCity,HubeiProvince | 108°58′58″ | 30°13′26″ | 1148 |
| CQ | JinziVillage,LongqiaoTown,Fengjie,ChongqingCity | 109°12′36″ | 30°39′03″ | 1237 |

Stable 2 Gradient elution condition of *R.officinale* Baill.

| Time | A:methanol：（%） | B：0.2% phosphoric acid（%） |
| --- | --- | --- |
| 0~5min | 15~30 | 85~70 |
| 5~10min | 30~50 | 70~50 |
| 10~15min | 50~58 | 50~42 |
| 15~25min | 58~60 | 42~40 |
| 25~36min | 60~90 | 40~10 |
| 35~40min | 90~100 | 10~0 |
| 40~45min | 100 | 0 |

Stable 3 Correlation analysis between effective ingredient content and soil factor content of *R. officinale* Baill

| components\  Soil factors | pH | Zn | Fe | Mn | Cu | TN | OM | AP | TP | TK | AK | NH₄⁺-N | NO₃⁻-N | SWC |
| --- | --- | --- | --- | --- | --- | --- | --- | --- | --- | --- | --- | --- | --- | --- |
| gallic acid | -0.921 | 0.123 | 0.872 | -0.456 | 0.789 | 0.845 | 0.832 | 0.901 | 0.867 | -0.654 | -0.789 | 0.912 | -0.876 | -0.432 |
| catechin | -0.845 | -0.234 | -0.765 | 0.321 | -0.678 | -0.712 | -0.704 | -0.812 | -0.745 | 0.543 | 0.654 | -0.801 | 0.765 | 0.298 |
| Sennoside B | 0.543 | 0.678 | -0.321 | 0.765 | -0.432 | -0.456 | -0.432 | 0.512 | -0.489 | -0.567 | -0.612 | 0.543 | -0.589 | 0.654 |
| Chrysophanol-8-O-β-D-glucopyranoside | -0.789 | 0.456 | 0.812 | -0.543 | 0.765 | 0.789 | 0.776 | 0.845 | 0.801 | -0.712 | -0.832 | 0.867 | -0.845 | -0.489 |
| emodin-8-glucoside | -0.654 | 0.321 | 0.712 | -0.432 | 0.654 | 0.678 | 0.665 | 0.732 | 0.698 | -0.589 | -0.701 | 0.745 | -0.712 | -0.376 |
| aloe-emodin | 0.432 | -0.567 | -0.654 | 0.789 | -0.298 | -0.321 | -0.309 | 0.456 | -0.376 | 0.432 | 0.489 | -0.432 | 0.465 | 0.543 |
| rhein | -0.701 | 0.376 | 0.745 | -0.489 | 0.712 | 0.732 | 0.721 | 0.789 | 0.754 | -0.654 | -0.765 | 0.801 | -0.776 | -0.432 |
| emodin | -0.589 | 0.298 | 0.654 | -0.376 | 0.589 | 0.612 | 0.601 | 0.665 | 0.632 | -0.543 | -0.654 | 0.698 | -0.665 | -0.321 |
| chrysophanol | 0.321 | -0.432 | -0.543 | 0.654 | -0.234 | -0.256 | -0.245 | 0.309 | -0.287 | 0.321 | 0.376 | -0.321 | 0.354 | 0.432 |
| physcion | -0.465 | 0.245 | 0.589 | -0.354 | 0.543 | 0.567 | 0.556 | 0.621 | 0.589 | -0.489 | -0.601 | 0.632 | -0.609 | -0.287 |

Stable 4 Pharmacological Effects of Ten Active Components in *R. officinale* Baill.

| Compound | Pharmacological effects |
| --- | --- |
| gallic acid | Alleviates inflammation and oxidative stress responses, and protects vascular endothelium and blood vessels |
| catechin | Antioxidant, anti-tumor, and cardiovascular protective effects |
| sennoside B | Laxative effect |
| chrysophanol-8-O-glucoside | Anti-inflammatory, analgesic, and antioxidant effects |
| emodin-8-O-glucoside | Anti-inflammatory, antioxidant, and cytoprotective effects |
| aloe-emodin | Antibacterial, skin-conditioning, anti-inflammatory, and anti-tumor effects |
| rhein | Including anti-inflammatory, anti-oxidative stress, anti-tumor, anti-fibrotic, lipid-regulating, hypoglycemic, antibacterial, and antiviral effects |
| emodin | Anti-tumor, anti-inflammatory, antiviral, antibacterial, and hepatorenal protective effects |
| chrysophanol | Neuroprotective, antitussive, antibacterial, and diuretic effects |
| physcion | Inhibits cerebral ischemic injury, and exerts neuroprotective and anti-tumor effects |
| gallic acid | Alleviates inflammation and oxidative stress responses, and protects vascular endothelium and blood vessels |
| catechin | Antioxidant, anti-tumor, and cardiovascular protective effects |
| sennoside B | Laxative effect |
